# Supplementary material for: Transcriptional changes in the mammary gland during lactation revealed by single cell sequencing of cells from human milk
Source: Nat Commun. 2022 Jan 28;13:562. doi: 10.1038/s41467-021-27895-0 (PMC8799659; doi:10.1038/s41467-021-27895-0)
Supplement: Supplementary file 1 — Supplementary Information [file 41467_2021_27895_MOESM1_ESM.pdf]

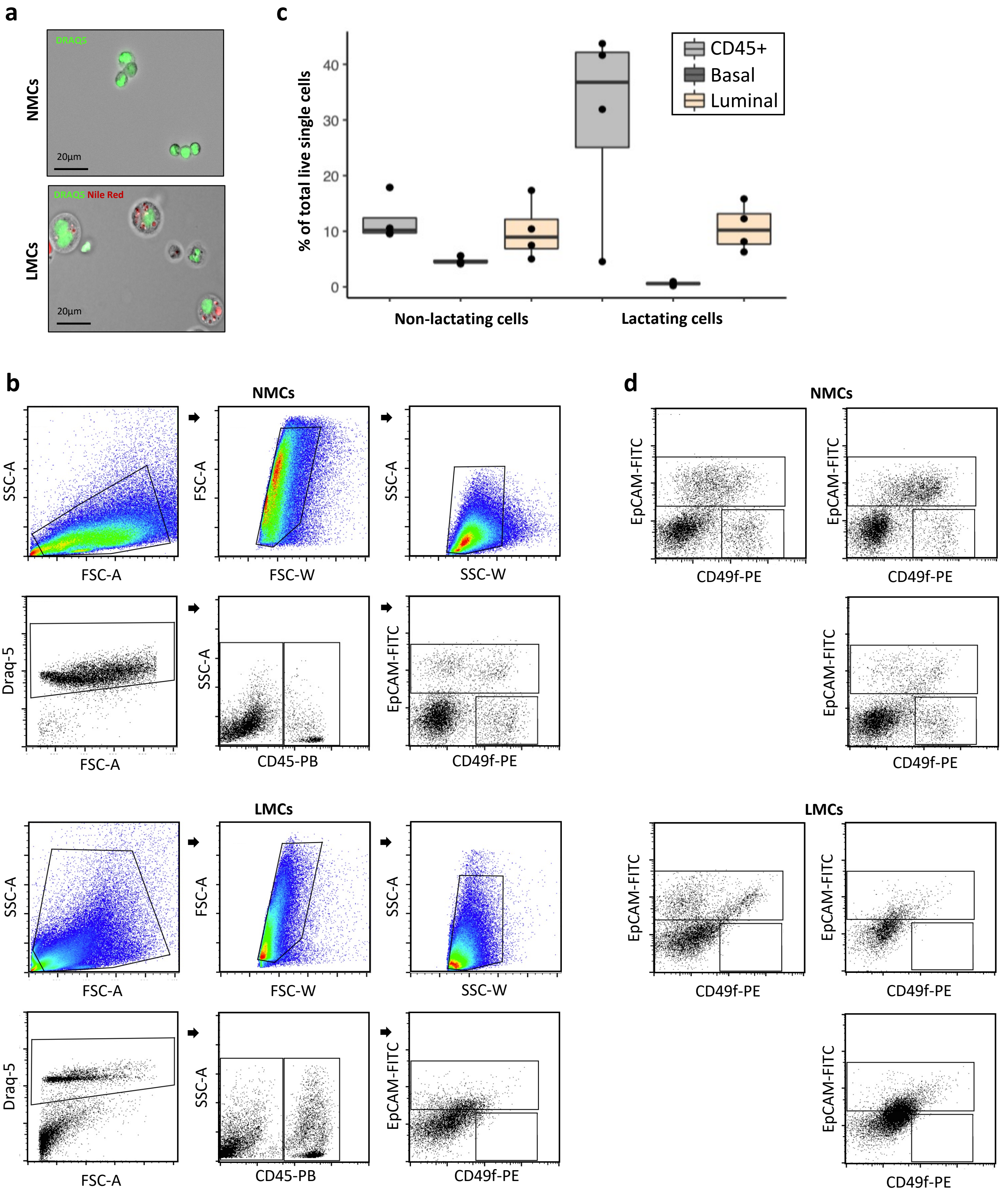

Supplementary Figure 1: Flow cytometry plots showing full gating strategy for the identification of mammary subpopulations in non-lactating mammary cell (NMC) or lactating mammary cell (LMC) samples. a) Differences in cell morphology between NMCs (above) and LMCs (below) could be visualized using light and fluorescence microscopy using nuclear stain Draq5 and neutral lipid stain Nile red, n>2. **b)** Representative full gating strategy shown for NMC (above) and LMC (below). **c)** Box and whisker plot summary of NMC (n=4) and LMC (n=4) that fall into the gates for single gated Draq5^{+} nucleated cells CD45^{+} immune cells, CD45^{-}/EpCAM^{-}/CD49f^{+} myoepithelial cells or CD45^{-}/EpCAM^{+} luminal cells. Centre line represents the median; box limits are the upper and lower quartiles; whiskers show 1.5x interquartile range and each point show the value for each sample. See Supplementary Table 1 for cell counts. **d)** Individual plots for remaining donors showing gated epithelial populations (using EpCAM and CD49f) from NMC (above) or LMC (below).

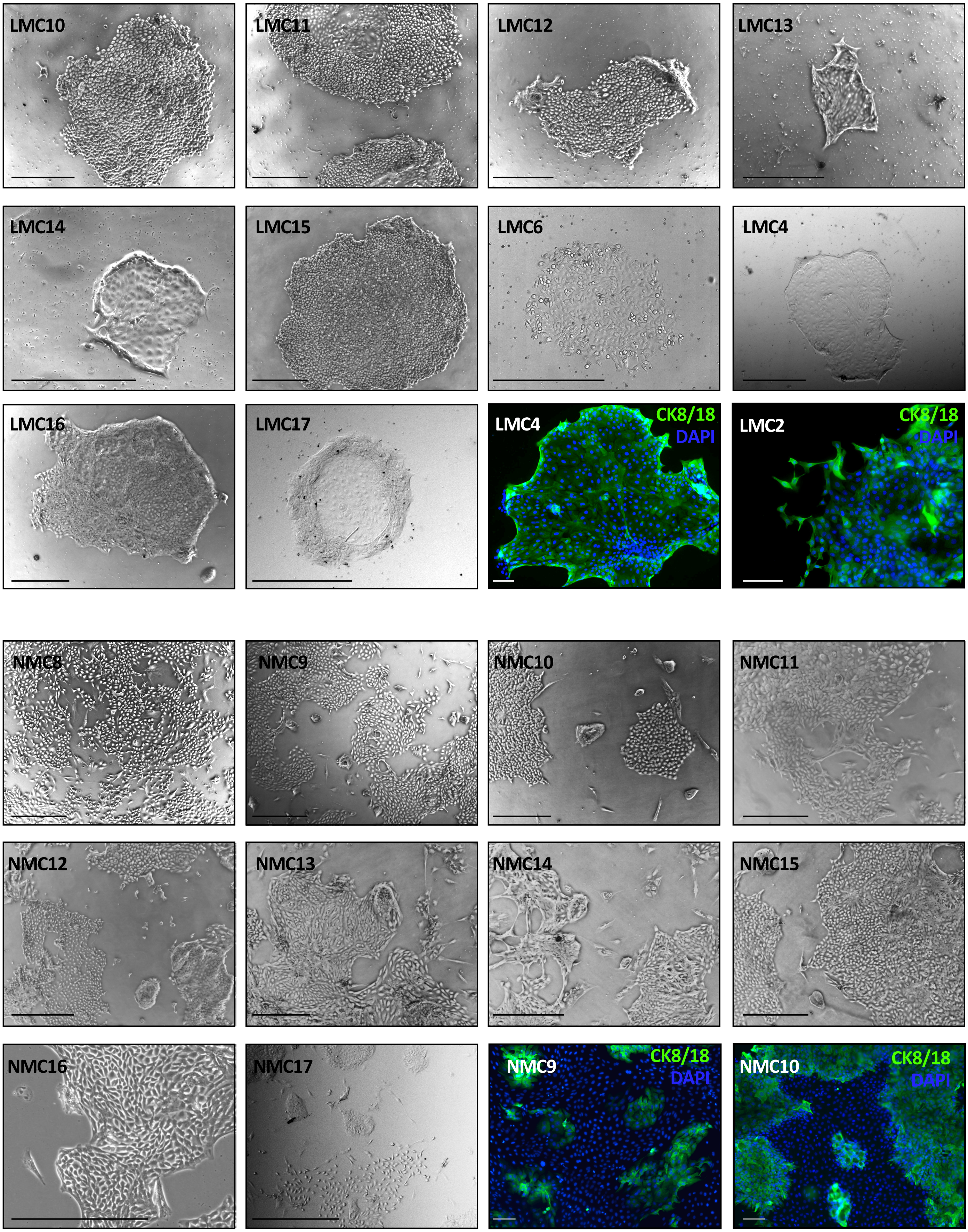

Supplementary Figure 2: **Phase and immunofluorescence images of 2D cultures of lactation associated mammary cells (LMC) and non-lactation associated mammary cells (NMC) derived from 10 different donors each.** Scale bar on phase contrast images represents 500 µm and scale bar on immunofluorescence is equal to 100 µm. Images were captured within 15 days of culturing.

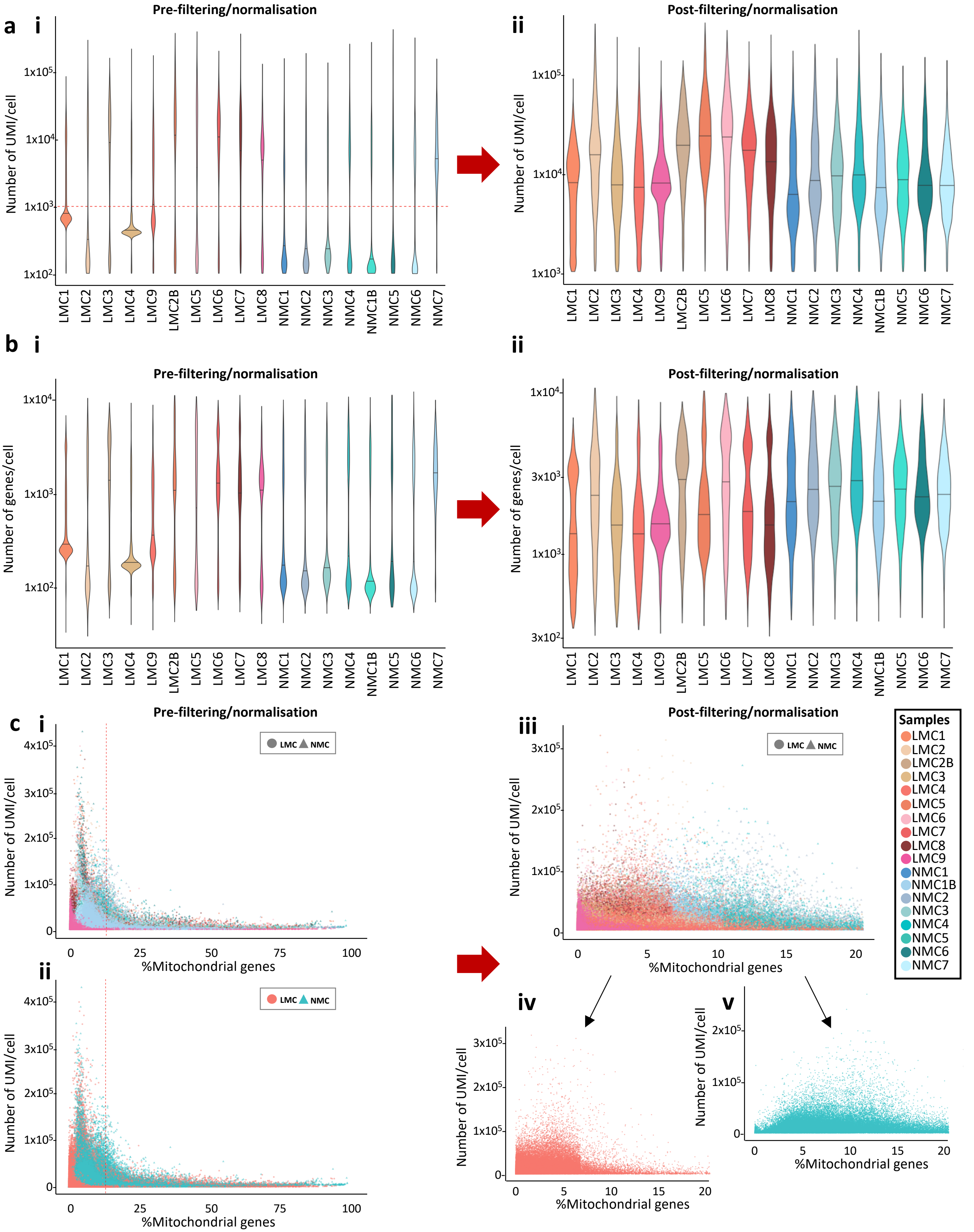

Supplementary Figure 3: **Overview of the scRNA-seq quality control measures undertaken on the lactating and non-lactating mammary cells (LMC and NMCs). a)** Violin plot of the unique molecular identifiers (UMIs) expressed per cell per sample **i)** before and **ii)** after filtering and normalisation. **b)** Violin plot of the genes expressed per cell per sample **i)** before and **ii)** after filtering and normalisation. **c)** Percentage of mitochondrial genes compared with number of UMI per cell before filtering/normalisation coloured either by **i)** sample or **ii)** state. Alternatively, plots showing the post-filtering/normalisation coloured either by **iii)** sample or showing **iv)** LMCs only or **v)** NMCs only). Red lines represent cut off thresholds used to filter the data.

a

Sample	Maternal age	Parity	Infant age	Batch	Fresh or Frozen	Total cells
NMC1*	33 yrs.	0	-	1	Freshly dissociated†	5339
NMC1B*	33 yrs.	0	-	3	Freshly dissociated†	12525
NMC2	41 yrs.	0	-	1	Freshly dissociated†	6943
NMC3	19 yrs.	0	-	1	Freshly dissociated†	6699
NMC4	47 yrs.	2	-	1	Freshly dissociated†	5685
NMC5	65 yrs.	1	-	2	Freshly dissociated†	8952
NMC6	54 yrs.	2	-	2	Freshly dissociated†	731
NMC7	54 yrs.	1	-	2	Freshly dissociated†	7840
LMC1	35 yrs.	1	3 months	1	Fresh	13102
LMC2*	43 yrs.	1	4 months	1	Viably frozen	2172
LMC2B*	43 yrs.	1	4 months	2	Fresh	7184
LMC3	27 yrs.	1	2 months	1	Fresh	5900
LMC4	35 yrs.	3	4 months	1	Fresh	5849
LMC5	44 yrs.	2	8 months	2	Viably frozen	2131
LMC6	39 yrs.	1	8 months	2	Viably frozen	2485
LMC7	39 yrs.	2	12 months	2	Viably frozen	6104
LMC8	39 yrs.	2	2 months	2	Viably frozen	6279
LMC9	33 yrs.	1	3 months	3	Fresh	4824

**Individual participant provided a sample that was included in two different batches* *†Samples were freshly dissociated from frozen fragments*

b

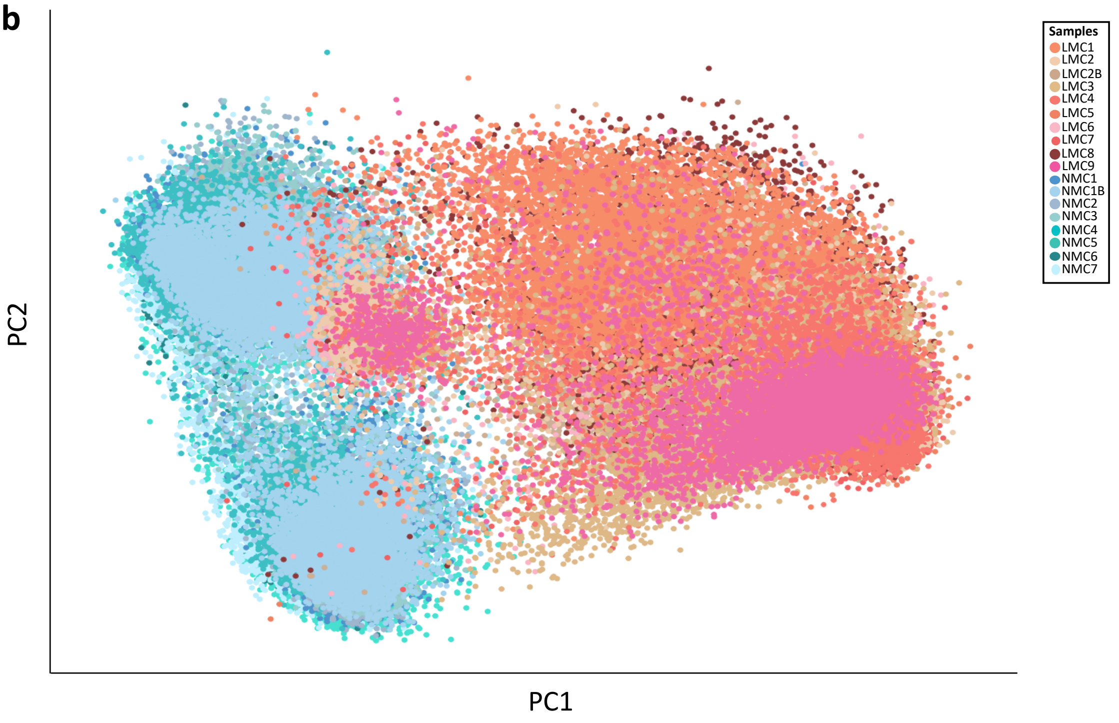

Supplementary Figure 4: Characteristics of donors and all samples used which have been normalised across batches used in this study. a) Table describing the demographics of each participant. **b)** Principal component (PC) analysis of all filtered and normalized cells revealed that the greatest variation along PC1 was due to samples coming from either lactating or non-lactating mammary cells (LMC or NMCs)

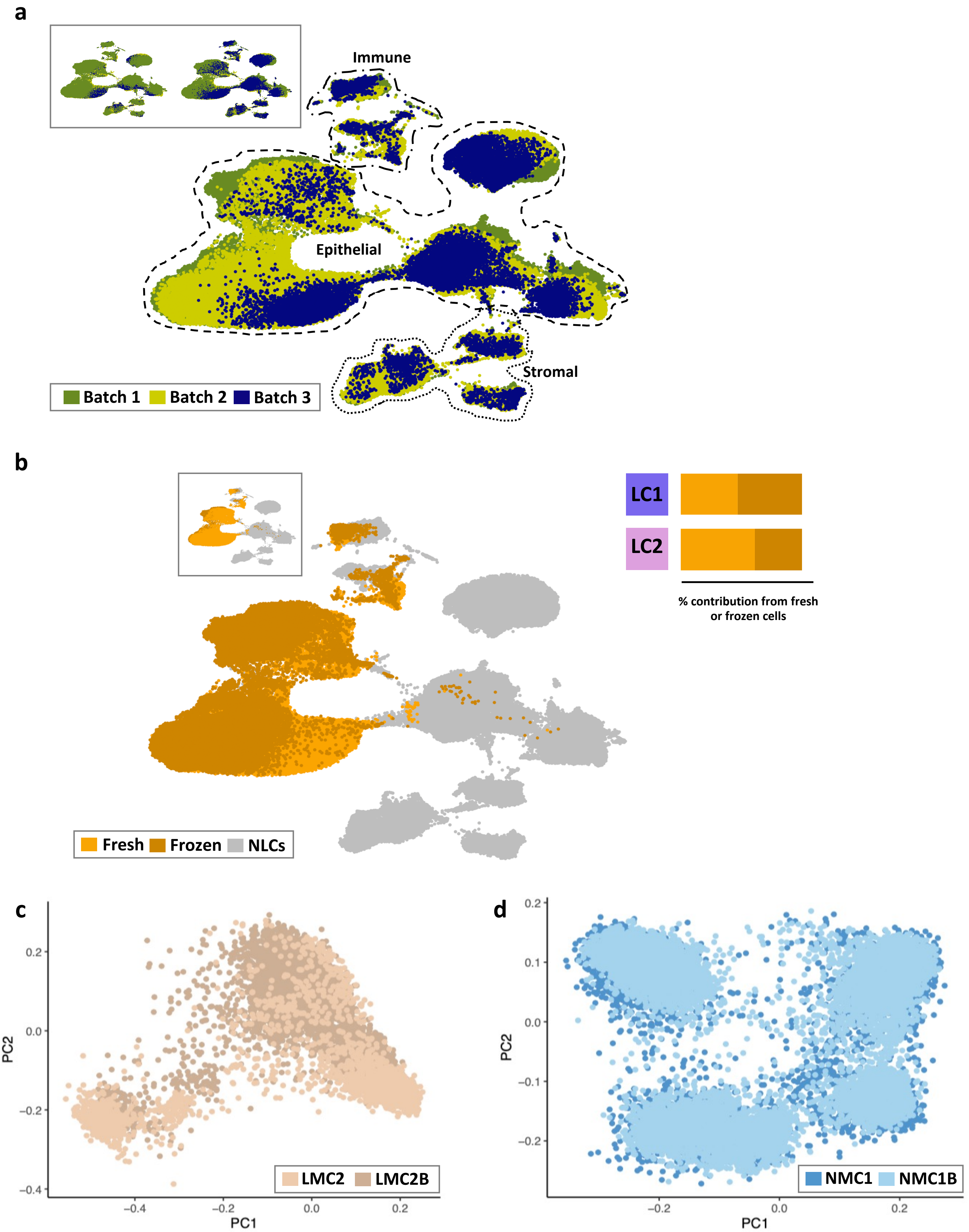

Supplementary Figure 5: **Batch or freezing effects did not significantly alter dimensional reduction plotting of cells after correction.** **a)** UMAP visualisation of cells coloured by batch reveal that cells from each batch contribute to all clusters. **b)** UMAP visualisation of lactating mammary cells (LMC) that were either processed from fresh milk or frozen down separately were found to overlap with no major cluster differences. The bar graphs represent the overall contribution of cells from fresh or frozen cells to clusters **LC1** and **LC2**. **c)** Cells from donor LMC2 that were sequenced fresh (LMC2) or after viable freezing (LMC2B) overlap in PCA analysis. **d)** Cells from donor NMC1 processed on two different days overlap.

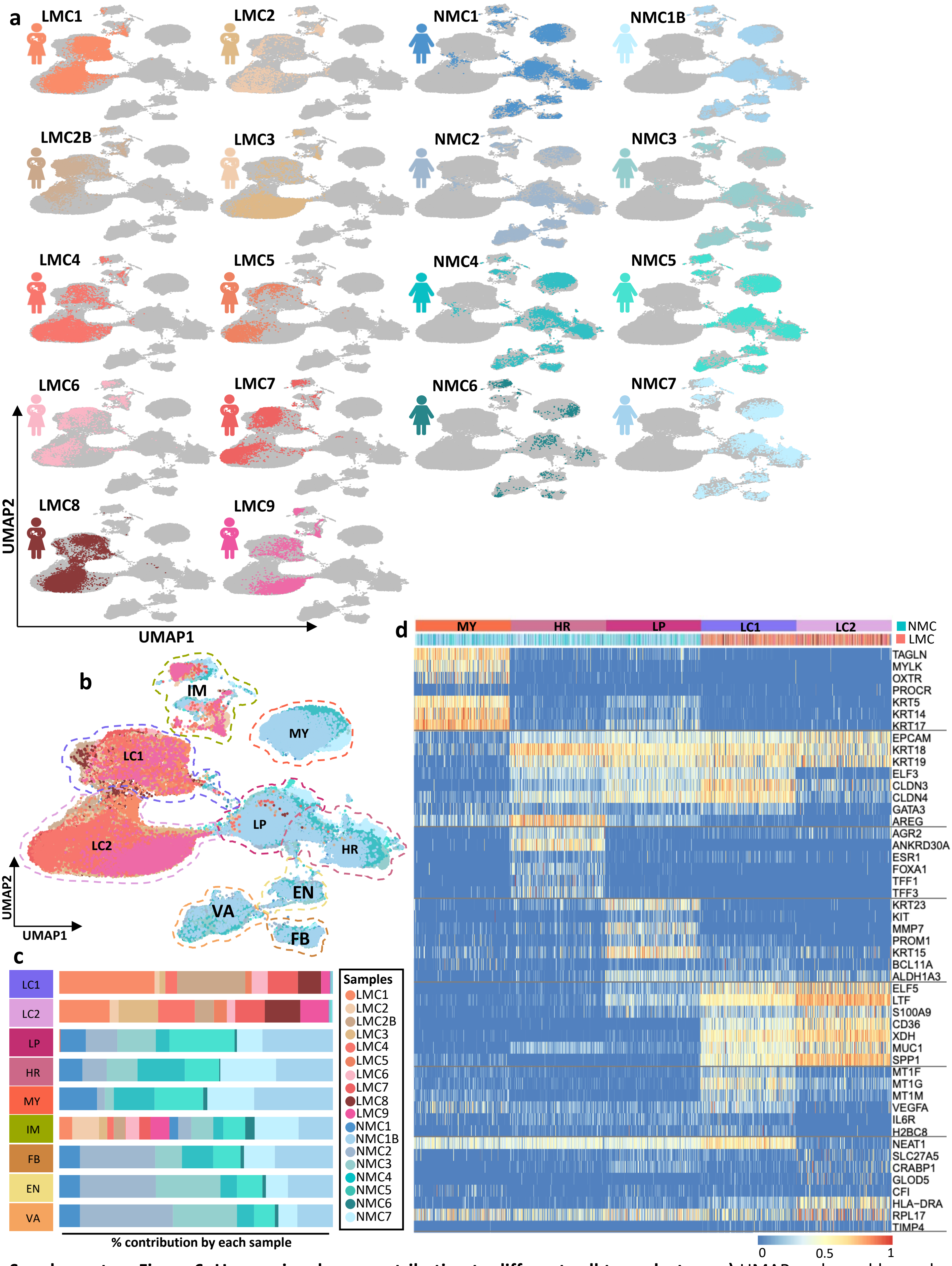

Supplementary Figure 6: **Uncovering donor contribution to different cell type clusters. a)** UMAPs coloured by each participant's cell contribution. **b)** Uniform manifold approximation and projection (UMAP) dimensional reduction of the mammary cells reveals distinct clusters arising from NMCs and LMCs, where cells are coloured by donor. **c)** Relative proportion of cells from each donor in each cell subtype cluster. **d)** Heatmap of key genes associated with each mammary cell subtype and identified in our clusters.

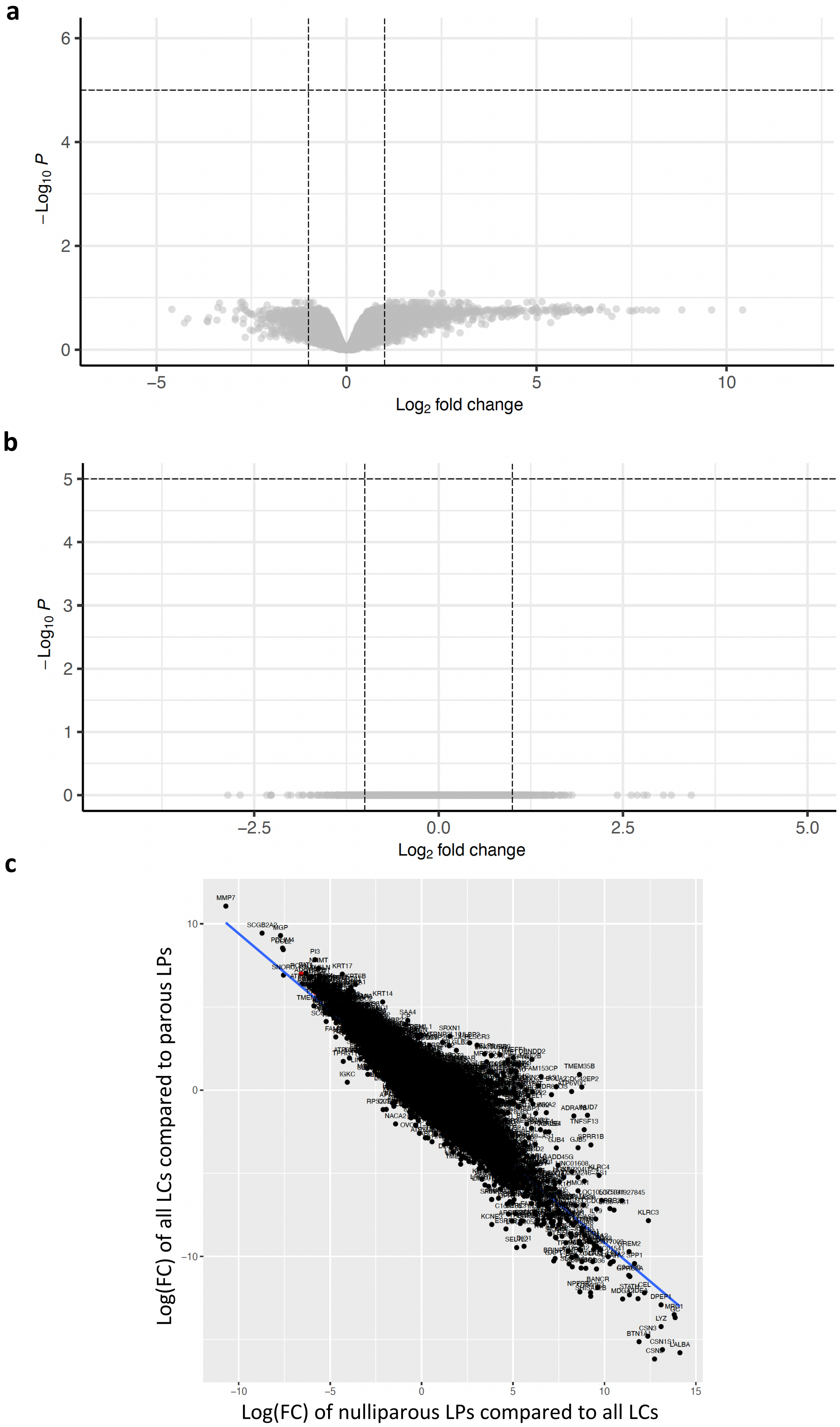

Supplementary Figure 7: **Examining the effect of parity on luminal cell gene expression. a)** Volcano plot of the genes differentially expressed between nulliparous and parous NMC luminal progenitors (LPs). **b)** Volcano plot of genes differentially expressed between uniparous and multiparous luminal cells (LCs) from milk. **c)** Contrasting fold changes of differentially expressed genes identified by comparing all LC LMCs to either nulliparous or parous LP NMCs.

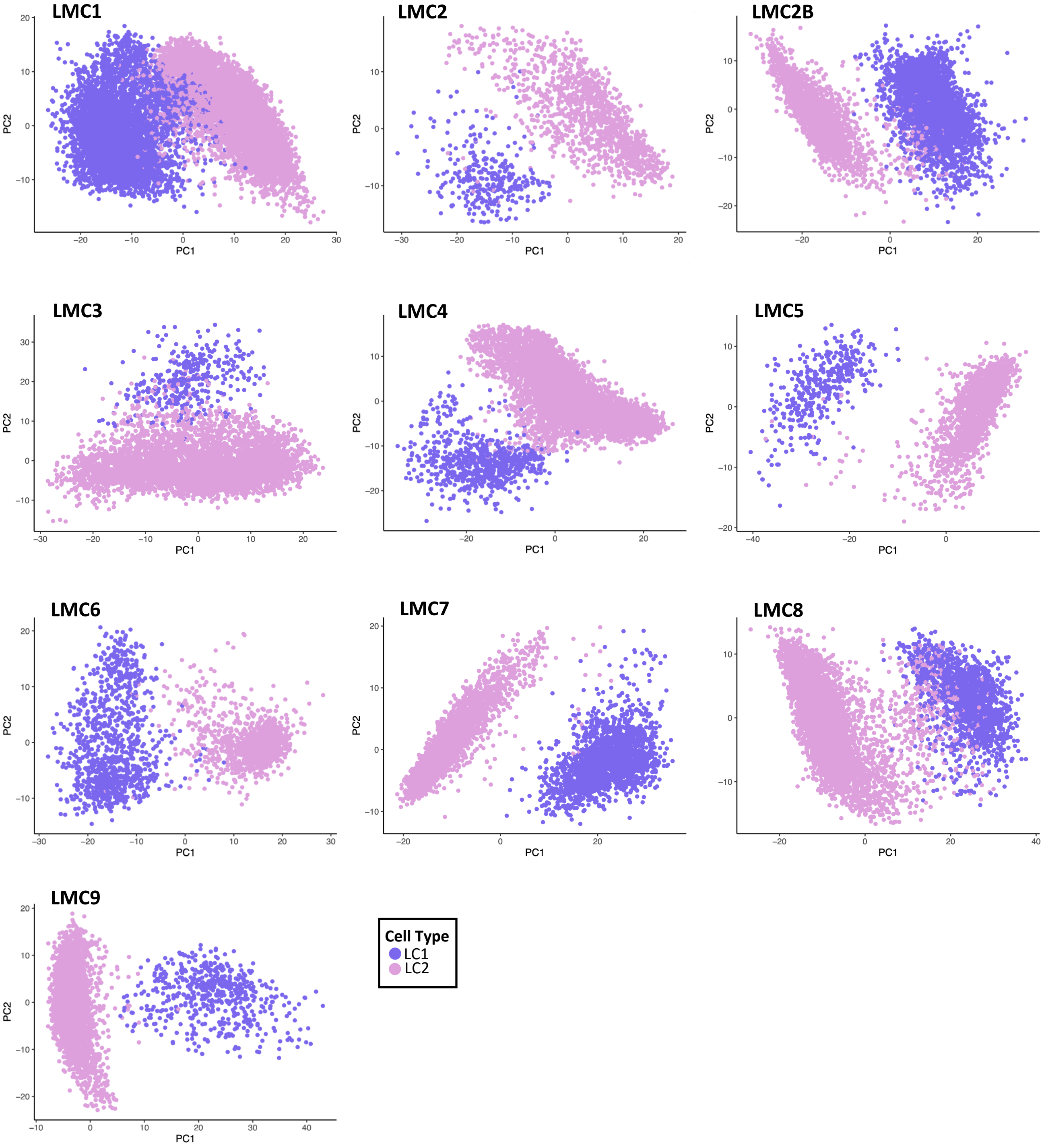

Supplementary Figure 8: **Principal component analysis of individual milk donor samples reveals cells separate into luminal clusters LC1 and LC2.**

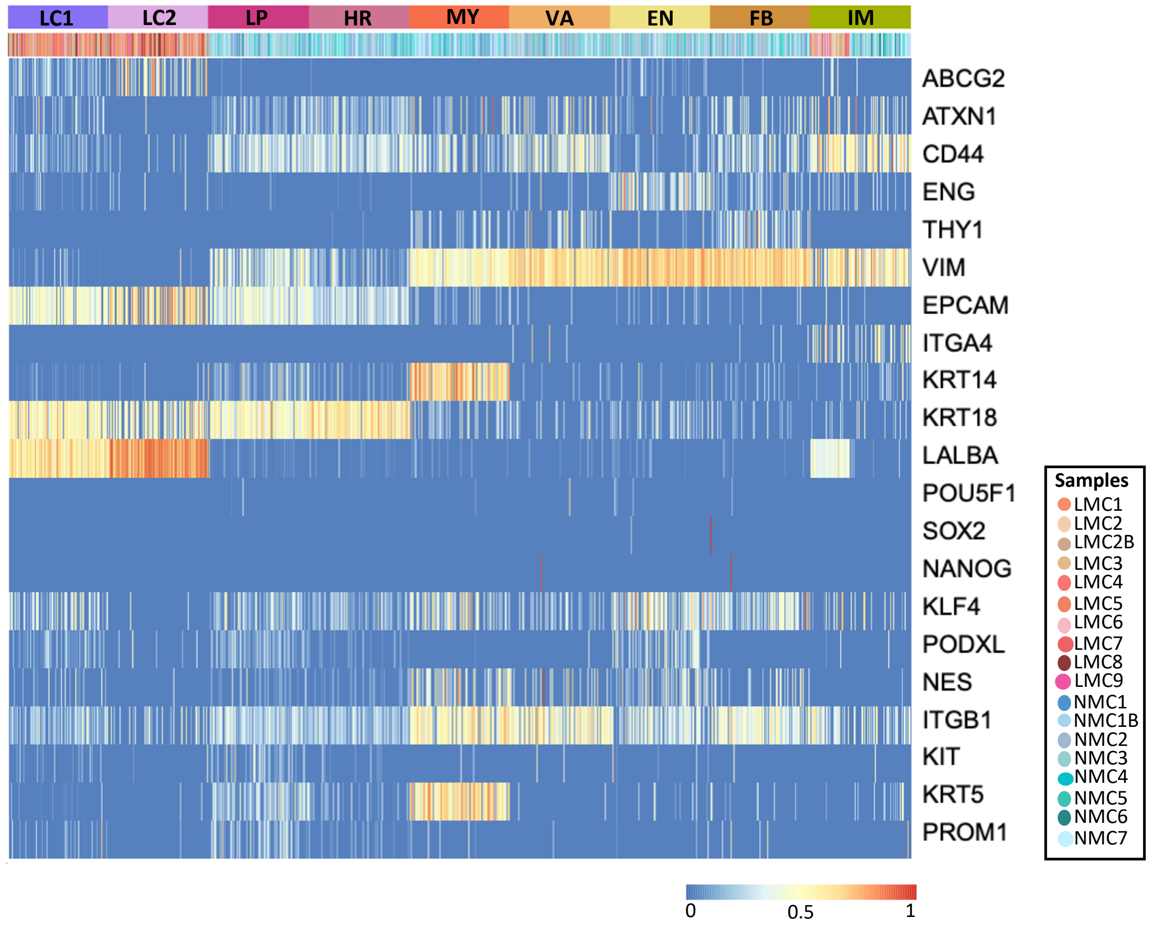

Supplementary Figure 9: Heatmap displaying the expression of key genes previously described in human milk cells across all lactation associated mammary cells (LMC) and non-lactation associated mammary cells (NMC) subtypes.

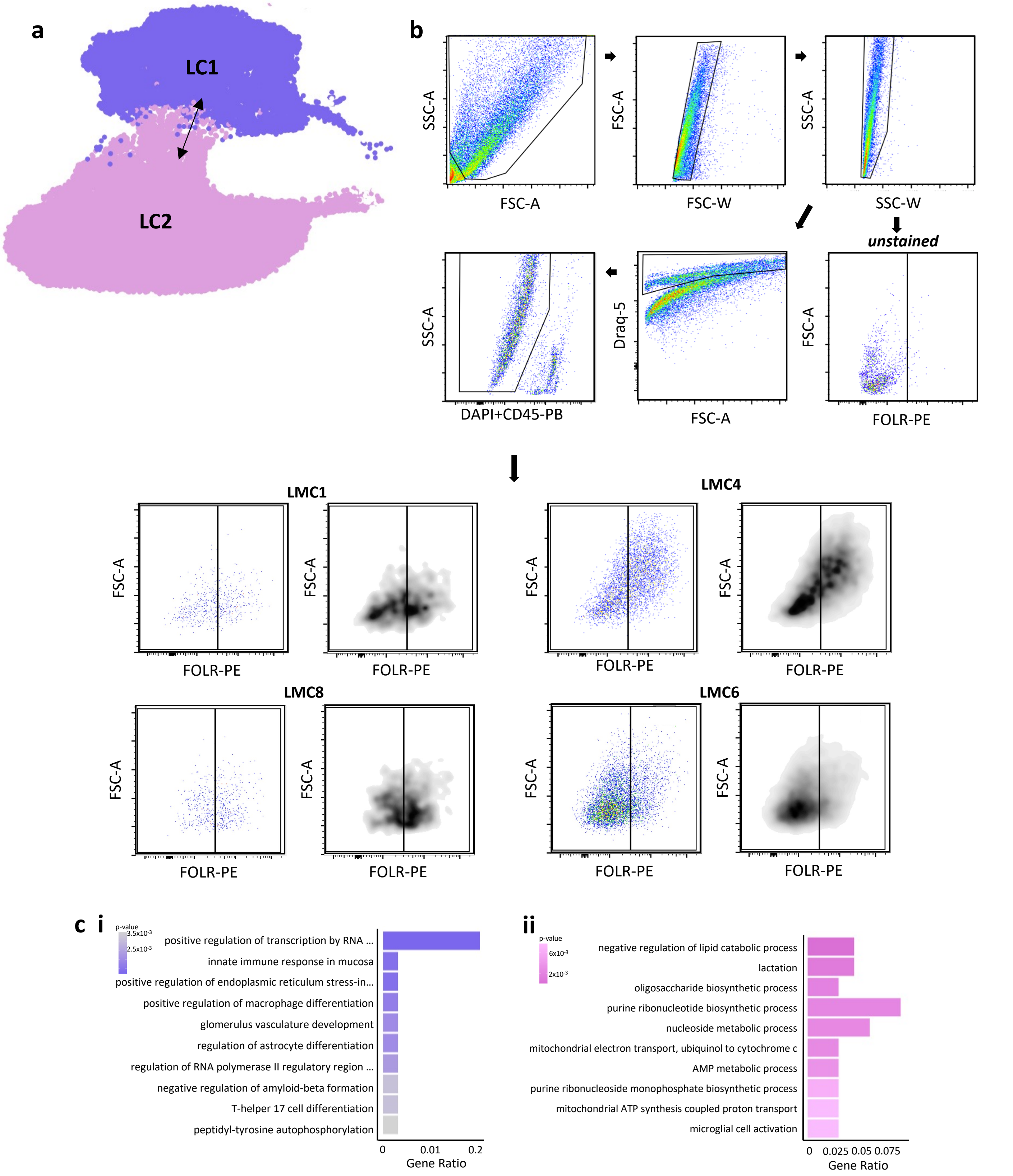

Supplementary Figure 10: **Exploring human milk lactation derived mammary cell (LMC) heterogeneity by comparing luminal clusters LC1 and LC2. a)** UMAP of LC1 and LC2 which are compared in this figure. **b)** Flow cytometry (FC) analysis of LMC1, LMC4, LMC6 and LMC8 separating LC1 and LC2 using folate receptor. **c)** The top 10 biological process gene ontology pathways that were associated with genes significantly differentially expressed that were either upregulated in either **i)** LC1 or **ii)** LC2 for a full list see Supplementary Datasets 2-3.

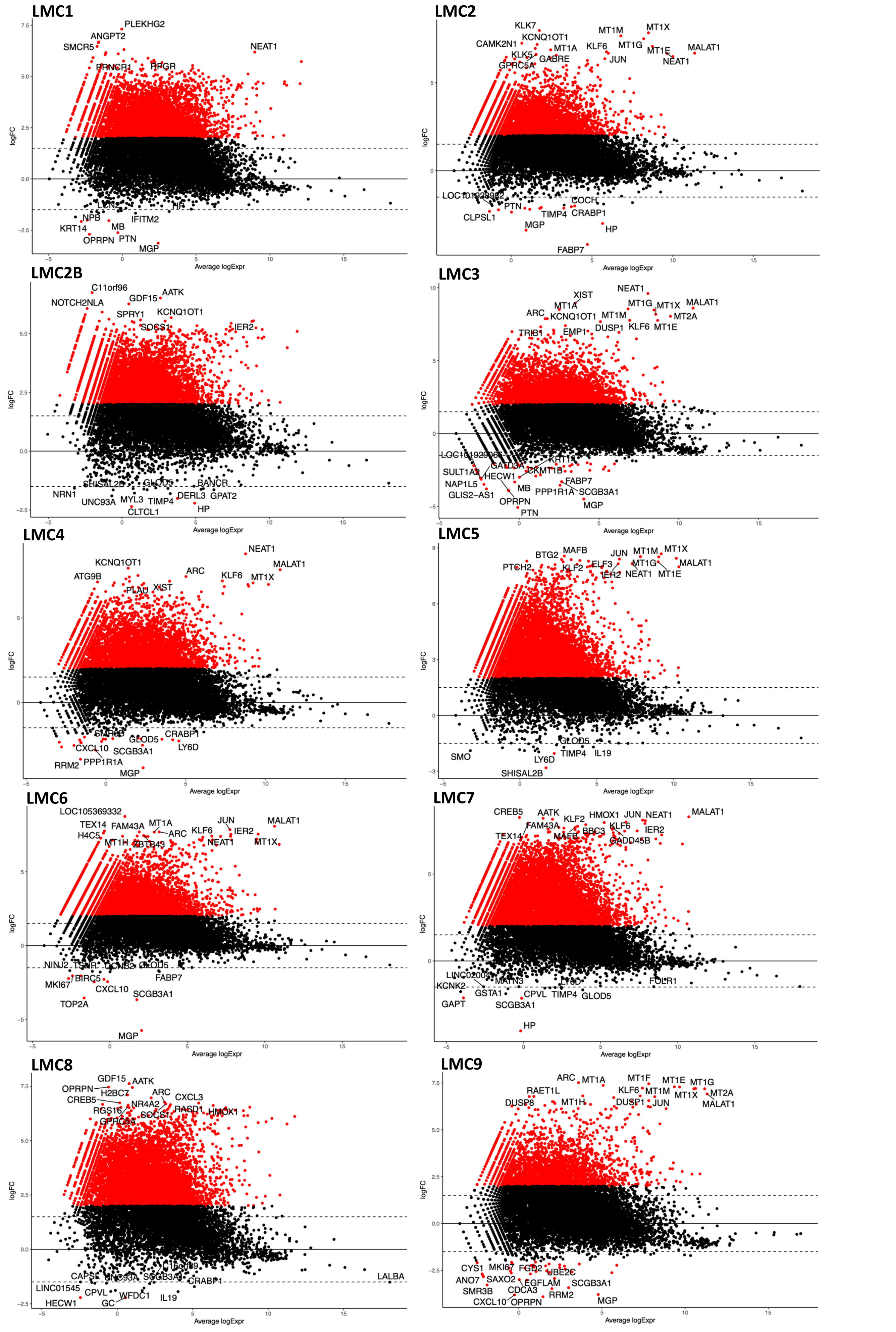

Supplementary Figure 11: MA plot of genes differentially expressed genes between luminal clusters LC1 and LC2 per milk cell sample.

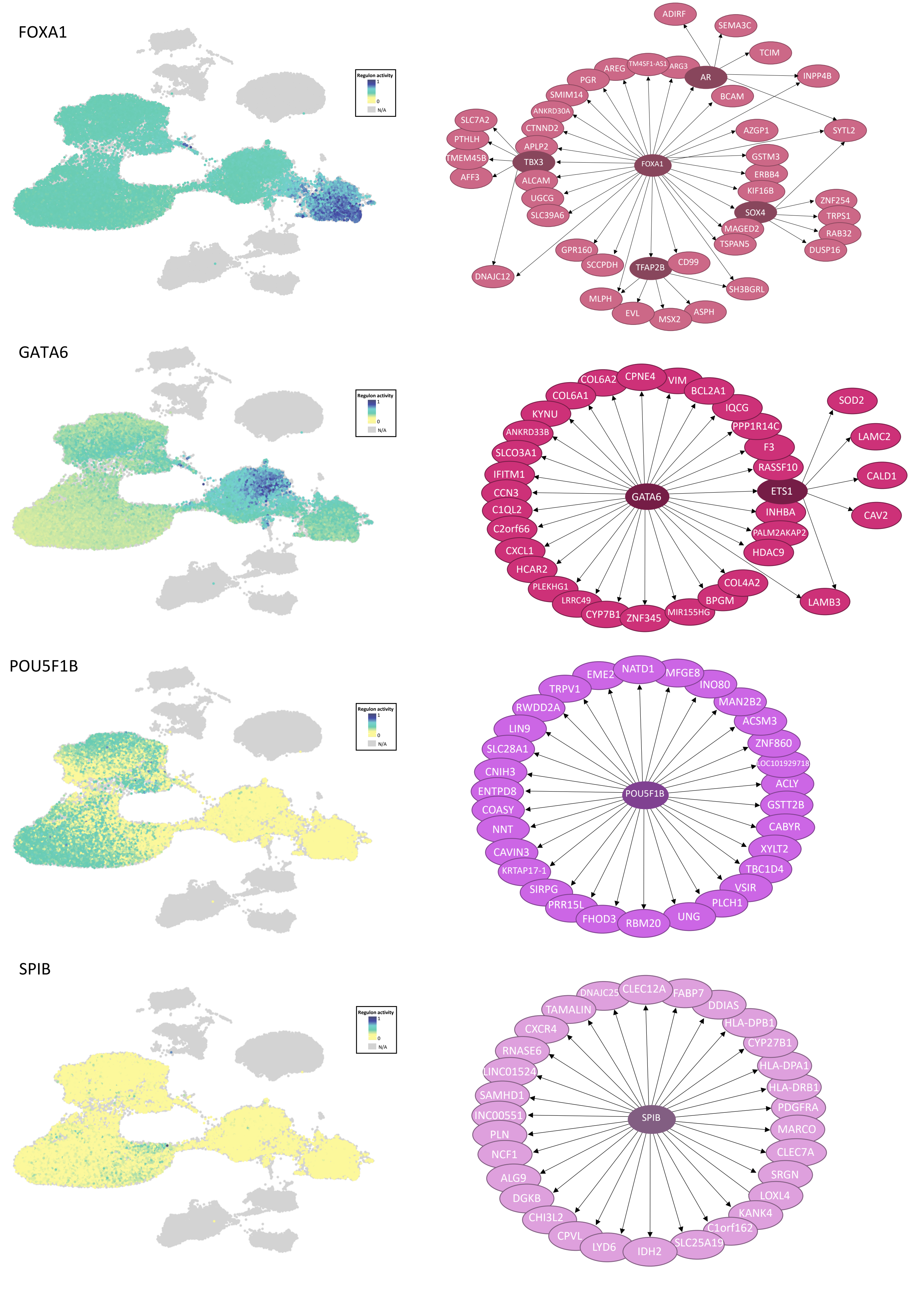

Supplementary Figure 12: UMAPs and schematic drawings of key regulons identified by analysing luminal cells from both milk and resting breast tissue.

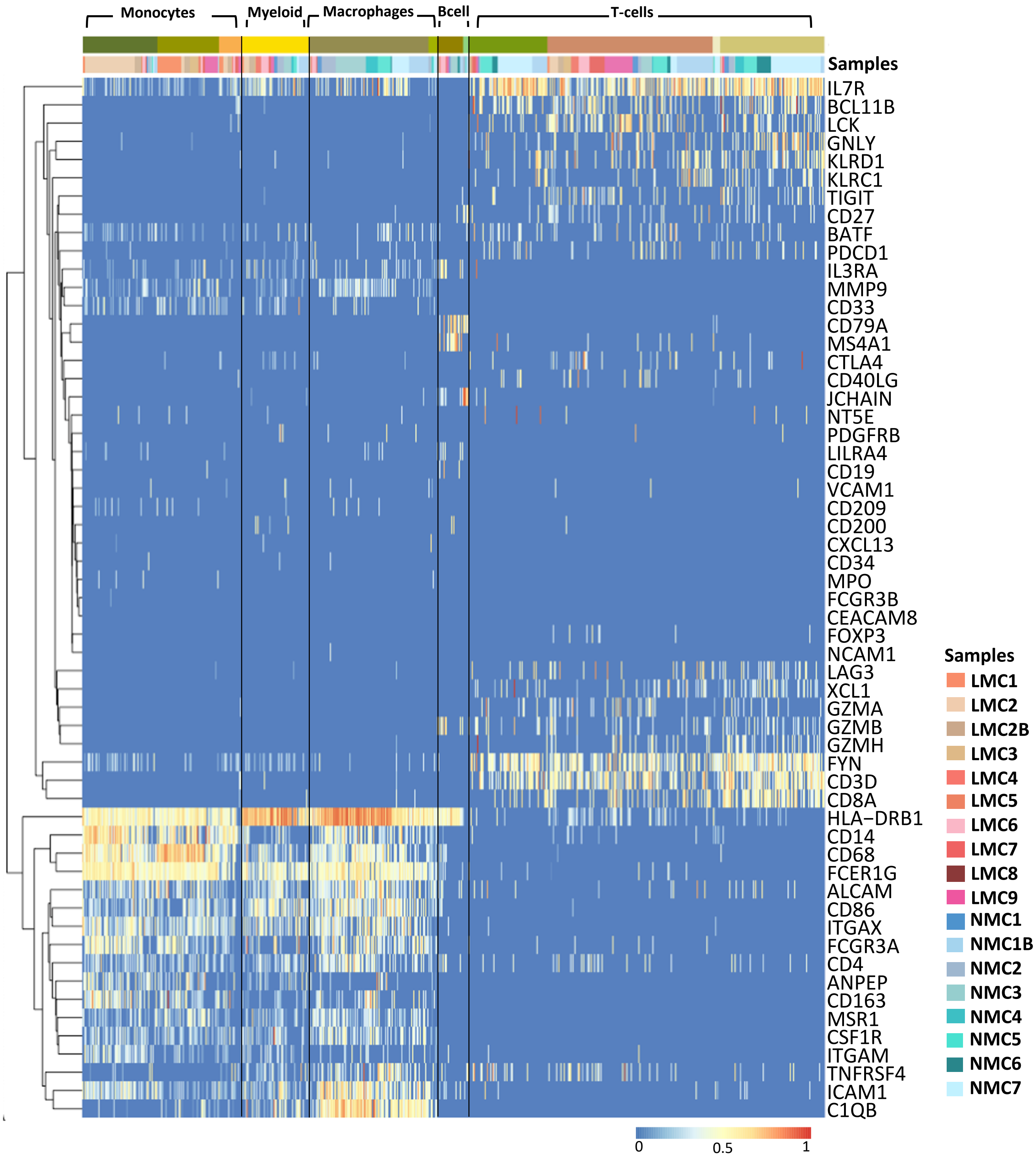

Supplementary Figure 13: Heatmap displaying the expression of genes characteristic of different immune cell subpopulations across both lactation and non-lactation associated mammary cells (LMC and NMCs). See Figure 3 for UMAP of cells coloured by matching sub-clusters.

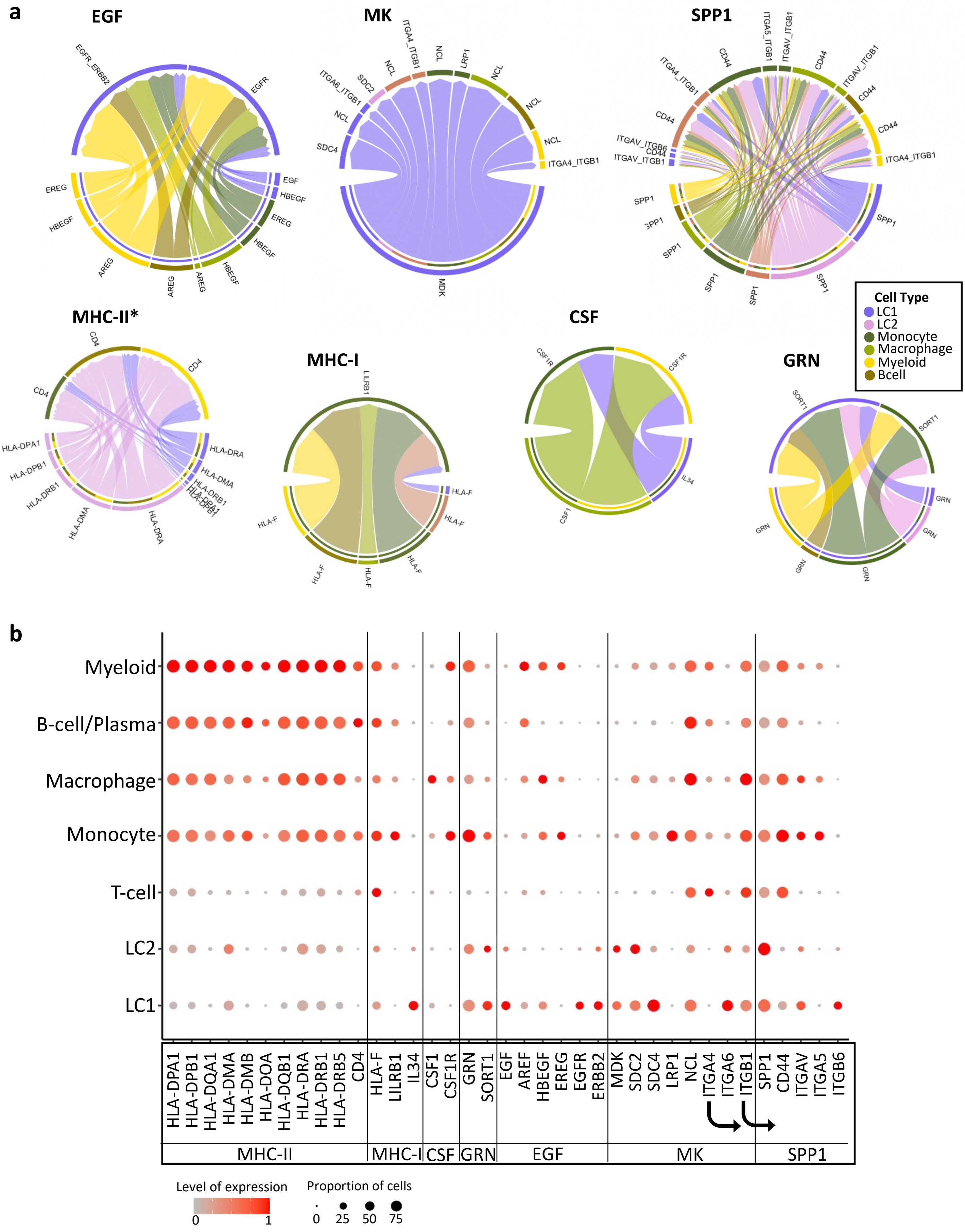

Supplementary Figure 14: Cell chat analysis a) Chord plots displaying an overview of all receptor-ligand signalling occurring between luminal and immune milk cells in the pathways of EGF, MK, SPP1, MHC-II, MHC-I, GRN and CSF. *The plot displaying the MHC-II pathways is only displaying signalling from luminal cells to immune cells, whereas all other plots are displaying all signalling occurring. **b)** Gene expression from all milk cell subtypes for each highlighted pathway. Arrows indicate that these genes are involved in both MK and SPP1 pathways.

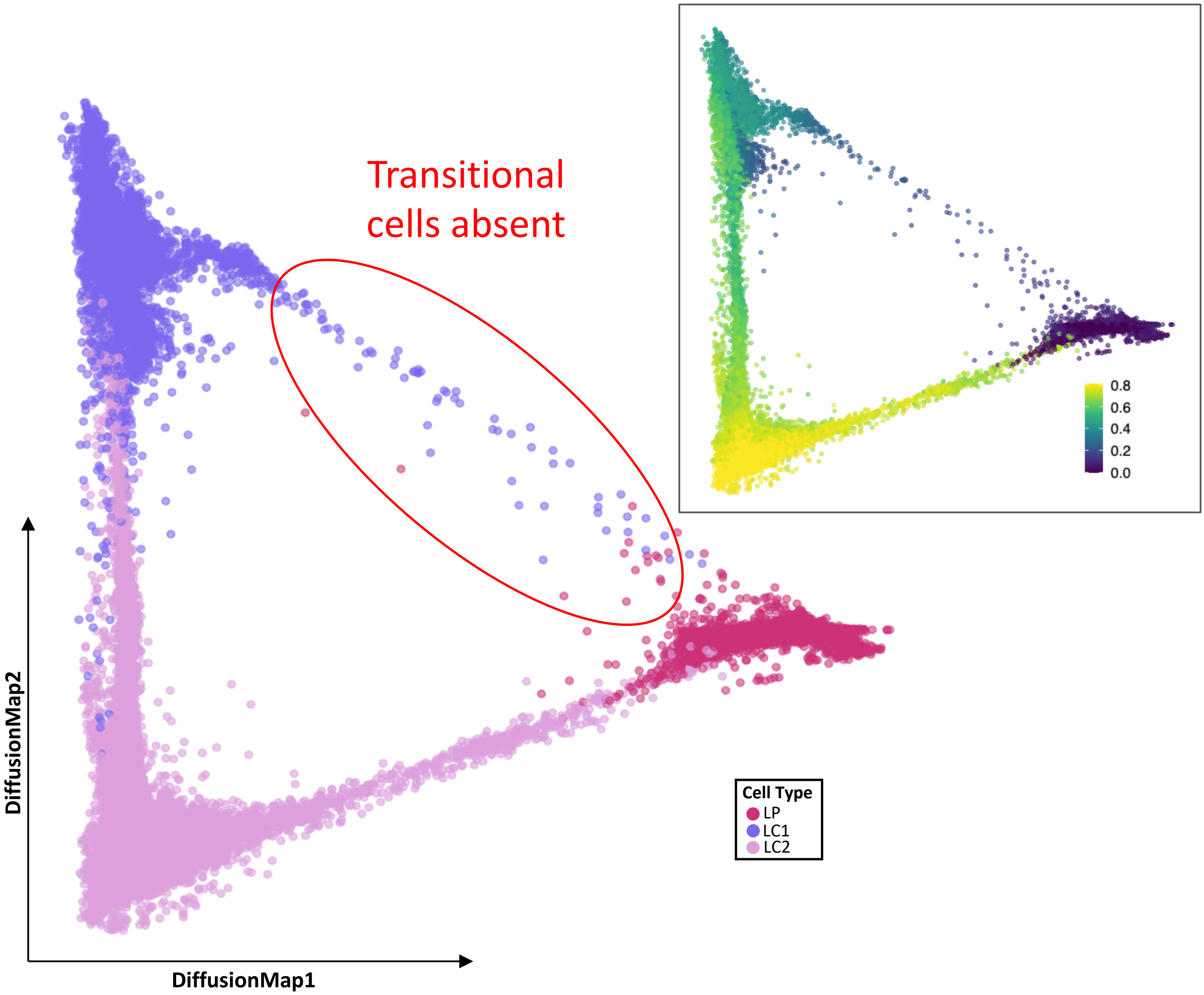

Supplementary Figure 15: Diffusion map analysis of luminal progenitor (LP) and luminal cells from the milk (LC1, LC2) reveal that a relationship exists between the cells, however pseudo time analysis (inset) demonstrates that intermediate cells are missing.

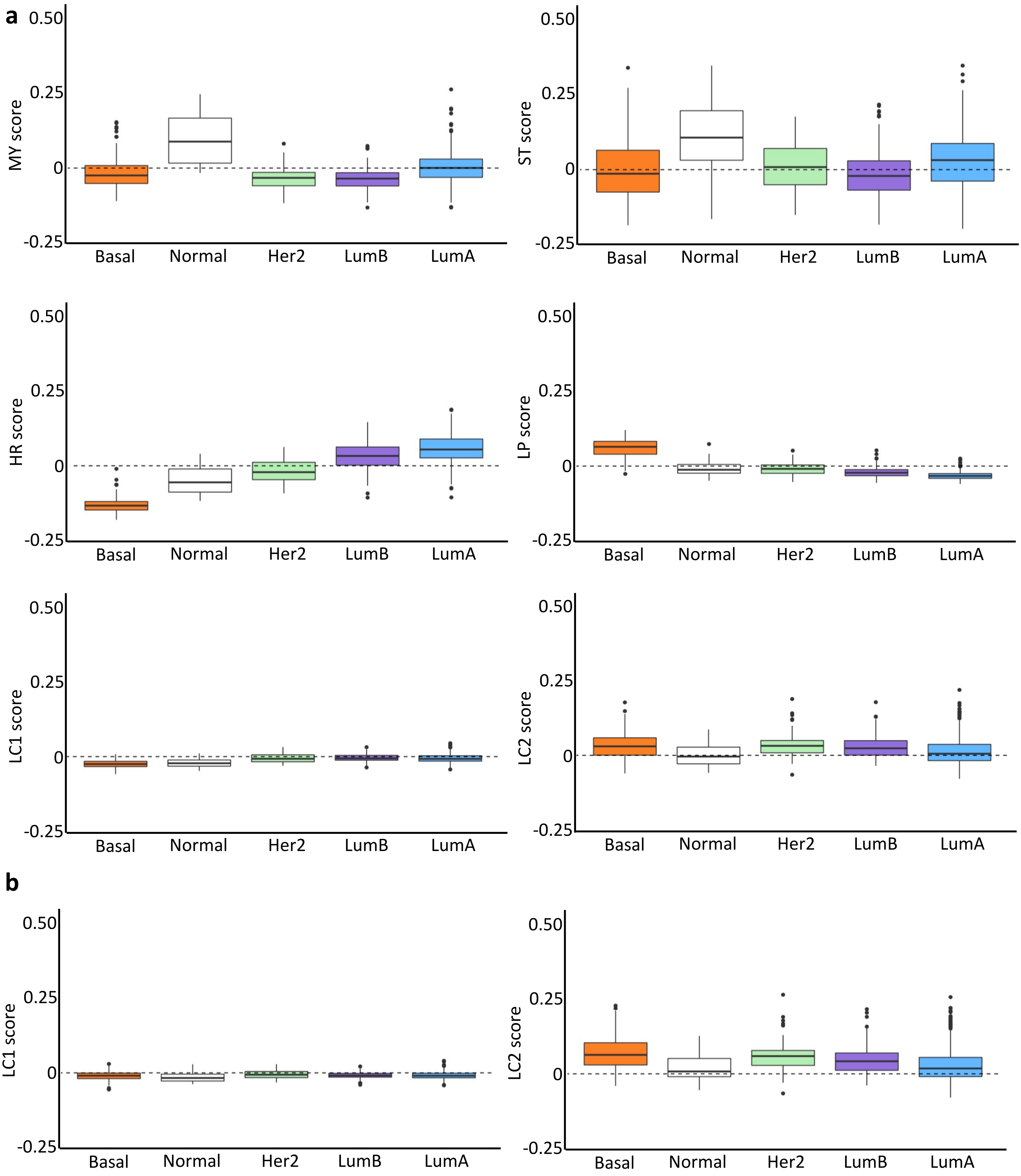

Supplementary Figure 16: Examining cell signature scores derived from our dataset for each cell type in breast tumour samples (n=1083) taken from The Cancer Genome Association (TCGA) that have been categorised using the molecular subtypes. a) Signatures for each cell type were highly expressed in either myoepithelial (MY), stromal (ST), hormone responsive (HR), luminal progenitor (LP), luminal cluster 1 (LC1) or luminal cluster 2 (LC2). Any gene found in more than one cluster was removed to ensure each gene signature was unique to the cell subtype. **b)** Plots display gene signatures from either LC1 and LC2, except in this case, genes shared with LPs were kept for both milk luminal signatures. In all box and whisker plots: centre line represents the median; box limits are the upper and lower quartiles; whiskers show 1.5x interquartile range and point show the outliers.

Supplementary Table 1: Flow cytometry (FC) counts for non-lactation associated mammary cells (NMC, n=4) or lactation associated mammary cells (LMC, n=4). See Supplementary Figure 1 for gating and summary.

	NMC-FC1	NMC-FC2	NMC-FC3	NMC-FC4	LMC-FC1	LMC-FC2	LMC-FC3	LMC-FC4
Total events	110357	161738	150000	300000	300000	300000	64777	300000
SSCA-FSCC gating	85049	120953	111332	238434	188462	116388	25901	161356
Single cells (gate 1)	81049	113687	103676	230037	174602	109795	23555	154312
Total single cells	77131	108380	98852	210849	156764	105759	22202	148130
$DRAQ5^+$	69677	98459	91353	192813	23444	39636	7385	44587
$CD45^+$	6798	17590	9657	18348	1064	12647	3229	18563
$CD45^-$	34608	67251	58030	108512	16036	17085	3752	21754
Basal	3098	4061	5103	8641	225	246	15	241
Luminal	7251	17071	6829	9685	3708	2489	601	5461
