# Supplementary material for: Transcriptional changes in the mammary gland during lactation revealed by single cell sequencing of cells from human milk
Source: Nat Commun. 2022 Jan 28;13:562. doi: 10.1038/s41467-021-27895-0 (PMC8799659; doi:10.1038/s41467-021-27895-0)
Supplement: Supplementary file 3 — Description of Supplementary Datasets [file 41467_2021_27895_MOESM3_ESM.docx]

**Additional Supplementary Files**

**Supplementary Dataset 1:** Differentially expressed genes (DEGs, derived using quasi-likelihood negative binomial generalized log-linear models) between luminal clusters **LC1** (downregulated) and **LC2** (upregulated). Significance is considered when the false discovery rate (FDR) corrected p-value is < 1x10^-8^.

**Supplementary Dataset 2:** Gene ontology (GO) terms for top differentially expressed genes (DEGs) upregulated in luminal cluster 1 (**LC1**) compared to **LC2**.

**Supplementary Dataset 3:** Gene ontology (GO) terms for top differentially expressed genes (DEGs) upregulated in luminal cluster 2 (**LC2**) compared to **LC1.**

**Supplementary Dataset 4**: All regulons by cell type identified by SCENIC analysis. High scores represent regulon specificity for the given luminal cell type.

**Supplementary Dataset 5:** All receptor-ligand pairs identified for the milk cell subtypes, calculated by determining the intercellular communication probability and then conducting permutation tests using CellChat.

**Supplementary Dataset 6:** Published mammary cell subpopulation scores.

**Supplementary Dataset 7:** Differentially expressed genes (DEGs, derived using quasi-likelihood negative binomial generalized log-linear models) between human milk luminal cluster cells (**LC**, upregulated) and non-lactating Luminal Progenitors (**LP**, downregulated). Significance is considered when the false discovery rate (FDR) corrected p-value is < 1x10^-8^.

**Supplementary Dataset 8:** Gene ontology (GO) terms for differentially expressed genes (DEGs) between luminal cells which were upregulated in luminal progenitor (**LP**) compared to luminal cluster (**LC**) cells.

**Supplementary Dataset 9:** Gene ontology (GO) terms for differentially expressed genes (DEGs) between luminal cells which were upregulated in luminal cluster (**LC**) cells compared to luminal progenitor (**LP**) cells.

**Supplementary Dataset 10**: Cell signatures for each cell type including: basal (**BA**), hormone responsive (**HR**), luminal cluster 1 and 2 (**LC1** and **LC2**), luminal progenitor (**LP**) or stromal (**ST**). Either with all overlapping genes removed (All_overlapping_removed=TRUE) or genes overlapping with LPs not removed (All_overlapping_removed=FALSE).
