# Supplementary material for: Transcriptional changes in the mammary gland during lactation revealed by single cell sequencing of cells from human milk
Source: Nat Commun. 2022 Jan 28;13:562. doi: 10.1038/s41467-021-27895-0 (PMC8799659; doi:10.1038/s41467-021-27895-0)
Supplement: Supplementary file 5 — Reporting Summary [file 41467_2021_27895_MOESM5_ESM.pdf]

nature research

Corresponding author(s): Alecia-Jane Twigger, Christina Scheel, Walid Khaled

Last updated by author(s): December 10, 2021

Reporting Summary

Nature Research wishes to improve the reproducibility of the work that we publish. This form provides structure for consistency and transparency in reporting. For further information on Nature Research policies, see our Editorial Policies and the Editorial Policy Checklist.

Statistics

For all statistical analyses, confirm that the following items are present in the figure legend, table legend, main text, or Methods section.

n/a	Confirmed	
☐	☒	The exact sample size (*n*) for each experimental group/condition, given as a discrete number and unit of measurement
☐	☒	A statement on whether measurements were taken from distinct samples or whether the same sample was measured repeatedly
☐	☒	The statistical test(s) used AND whether they are one- or two-sided<br>*Only common tests should be described solely by name; describe more complex techniques in the Methods section.*
☐	☒	A description of all covariates tested
☐	☒	A description of any assumptions or corrections, such as tests of normality and adjustment for multiple comparisons
☐	☒	A full description of the statistical parameters including central tendency (e.g. means) or other basic estimates (e.g. regression coefficient) AND variation (e.g. standard deviation) or associated estimates of uncertainty (e.g. confidence intervals)
☐	☒	For null hypothesis testing, the test statistic (e.g. *F*, *t*, *r*) with confidence intervals, effect sizes, degrees of freedom and *P* value noted<br>*Give P values as exact values whenever suitable.*
☒	☐	For Bayesian analysis, information on the choice of priors and Markov chain Monte Carlo settings
☐	☒	For hierarchical and complex designs, identification of the appropriate level for tests and full reporting of outcomes
☒	☐	Estimates of effect sizes (e.g. Cohen's *d*, Pearson's *r*), indicating how they were calculated

Our web collection on statistics for biologists contains articles on many of the points above.

Software and code

Policy information about availability of computer code

Data collection	All details are provided in the methods section of the manuscript. Briefly, Flow cytometry data was acquired using FACSDivaTM 6.0 Software. Sequencing was performed on a NovaSeq6000 S2 (batches 1&2) and Illumina HiSeq 4000 (batch 3).
Data analysis	All details are provided in the methods section of the manuscript and R scripts will be made publicly available through Github (https://github.com/aleciajane/LactatingMammaryCells.git). Briefly, sc-RNA sequencing data was aligned and initial processing was done using: Cell Ranger Single-Cell Suite version 3.0.2 (for batches 1 &2) or version 2.1.1 (batch 3) provided by 10x Genomics. Data analysis was conducted in R (versions 3.5.3-4.1.0, versions specified for each script on github) downloaded from https://cran.r-project.org/. Aside from base packages, many packages were downloaded from Bioconductor (https://www.bioconductor.org/) through R including: DropletUtils, scater, BiocSingular version 1.6.0, umap version 0.2.7.0, scran version 1.16.0, ggplot2, pheatmap, edgeR version 3.34.3, EnhancedVolcano, Seurat version 4.0.2-3, Cellchat version 1.1.0. and TCGAbiolinks version 2.20.0. Flow cytometry data was analyzed using FlowJo_V10 Software (FlowJo LLC, Ashland, U.S.)

For manuscripts utilizing custom algorithms or software that are central to the research but not yet described in published literature, software must be made available to editors and reviewers. We strongly encourage code deposition in a community repository (e.g. GitHub). See the Nature Research guidelines for submitting code & software for further information.

Data

Policy information about availability of data

All manuscripts must include a data availability statement. This statement should provide the following information, where applicable:

- Accession codes, unique identifiers, or web links for publicly available datasets
- A list of figures that have associated raw data
- A description of any restrictions on data availability

The authors declare that all data supporting the findings of this study are available within the article and its supplementary information files or from the corresponding authors upon request. Each batch of the RNA sequencing data has been deposited in the Array Express database and can be retrieved by the following access IDs: E-MTAB-9841 (Batch 1), E-MTAB-10855 (Batch 2) and E-MTAB-10885 (Batch 3) which will be released upon publication. A user-friendly website is available at http://bioinf.stemcells.cam.ac.uk:3838/khaled_wUFt1bHfmC/twigger/ to enable Data exploration. All computational analyses were performed in R (Versions 3.5.3-4.1.0) using standard functions unless otherwise indicated. TCGA data was accessed through the R package TCGAbiolinks version 2.20.0.

Field-specific reporting

Please select the one below that is the best fit for your research. If you are not sure, read the appropriate sections before making your selection.

[x] Life sciences   [ ] Behavioural & social sciences   [ ] Ecological, evolutionary & environmental sciences

For a reference copy of the document with all sections, see nature.com/documents/nr-reporting-summary-flat.pdf

Life sciences study design

All studies must disclose on these points even when the disclosure is negative.

Sample size	Sample size was limited by availability.
Data exclusions	No data was excluded from analysis
Replication	All experiments were conducted in 2-10 different biological replicates, as described in the manuscript.
Randomization	Randomization was not necessary as we were not testing different experimental conditions.
Blinding	Blinding was not relevant for this study as no treatments were provided to the participants.

Reporting for specific materials, systems and methods

We require information from authors about some types of materials, experimental systems and methods used in many studies. Here, indicate whether each material, system or method listed is relevant to your study. If you are not sure if a list item applies to your research, read the appropriate section before selecting a response.

Materials & experimental systems

n/a	Involved in the study	
[ ]	[x]	Antibodies
[x]	[ ]	Eukaryotic cell lines
[x]	[ ]	Palaeontology and archaeology
[x]	[ ]	Animals and other organisms
[ ]	[x]	Human research participants
[x]	[ ]	Clinical data
[x]	[ ]	Dual use research of concern

Methods

n/a	Involved in the study	
[x]	[ ]	ChIP-seq
[ ]	[x]	Flow cytometry
[x]	[ ]	MRI-based neuroimaging

Antibodies

Antibodies used	CD45-PB (V450, catalogue number: 560368, BD, Heidelberg, Germany), EpCAM-FITC (catalogue number:GTX79849-100, GeneTex), CD49f-PE (catalogue number: 555736,BD), anti-human Folate Receptors α and β (FR-αβ)-PE (catalogue number: 391805, Biolegend), CD45-PB (catalogue number: 304021, Biolegend, California, U.S.), CK8/18 antibody (DLN-010750, Dianova, Castelldefels, Spain), Anti-Mouse IgG Alexa Fluor 488 Donkey Anti-Mouse IgG (A21202,ThermoFisher Scientific,Waltham, U.S.)
Validation	FACS antibodies used were described previously in Linnemann et al. 2015. Staining pattern of CD45 was the same across the different antibodies. Negative control is shown for FR-αβ. Staining for CK8-18 was not observed in all cells and when it was it was specific to the correct region of the cells (intermediate filaments). All pictures taken was normalized to a secondary only control.

Human research participants

Policy information about studies involving human research participants

Population characteristics	Participant details for scRNA-seq data are described in Supplementary Figure 2A. Briefly breast tissue was collected from 3 nulliparous females and 4 parous female (parity of 1-2) with ages ranging from 19-65 years old. Milk samples from 5 uniparous females and 4 multiparous female (parity of 2-3) ranging from 27-43 years old.
Recruitment	Human milk donors were recruited from Pippagina English Prenatal and Postnatal classes or through the Helmholtz Zentrum München. Selection bias exists, where the recruitment targeted English speaking Munich residents who either attend classes or are affiliated with Helmholtz Center Munich. Human breast tissue donors were recruited from Nymphenburg Clinic for Plastic and Aesthetic Surgery and selection bias exists for women seeking aesthetic breast reduction surgery.
Ethics oversight	Non-lactating human breast tissue was donated by participants who provided informed consent who were undergoing elective aesthetic reduction mammoplasty at the Nymphenburg Clinic for Plastic and Aesthetic Surgery in accordance with the regulations of the ethics committee of the Ludwig-Maximilian University, Munich, Germany (proposal 397-12).<br><br>Human milk donors were recruited from Pippagina English Prenatal and Postnatal classes or through the Helmholtz Zentrum München in accordance with regulations of the ethics committee of the Ludwig-Maximilian University, Munich, Germany (proposal 17–715).

Note that full information on the approval of the study protocol must also be provided in the manuscript.

Flow Cytometry

Plots

Confirm that:

- [x] The axis labels state the marker and fluorochrome used (e.g. CD4-FITC).
- [x] The axis scales are clearly visible. Include numbers along axes only for bottom left plot of group (a 'group' is an analysis of identical markers).
- [x] All plots are contour plots with outliers or pseudocolor plots.
- [x] A numerical value for number of cells or percentage (with statistics) is provided.

Methodology

Sample preparation	This has been described in the manuscript in detail, briefly isolated cells were stained with CD45-PB (dilution of 1:100), EpCAM-FITC (dilution of 1:10) and CD49f-PE (dilution of 1:20). After incubation, stained MESs were diluted in MECGM and filtered through 35 µm cell strainer caps of round-bottom tubes (Corning, Corning, U.S.). Prior to analysis, cells were stained with with DRAQ5 to a final concentration 1µM. Similarly, milk cells were stained for CD45-PB(1:100) and FOLR-PE(1:40) for 45 minutes. Following this, cells were washed and filtered and DAPI and DRAQ5 were incubated with the cells for 10 minutes prior to analysis. In both cases, small volumes of cells from each sample were mixed prior to analysis and used as comparison and control.
Instrument	FACSAriaTM III cell sorter, BD Biosciences or BD LSRFortessa machine, BD Biosciences
Software	FlowJo_V10 Software (FlowJo LLC, Ashland, U.S.)
Cell population abundance	No sorts were performed for this study
Gating strategy	Initial FSC-A vs SSC-A, FSC-A vs FSC-A and SSC-W vs SSC-A gating was done depending on sample origin (either milk or breast tissue) but all subsequent analysis (e.g. FSC-A vs CD45) of the samples was done using the gating strategies across all samples. Gating strategies are shown in Supplementary Figure 1 and 7.

- [x] Tick this box to confirm that a figure exemplifying the gating strategy is provided in the Supplementary Information.
